# EXPLORING THE MULTIDIMENSIONAL BIOPSYCHOSOCIAL PATHWAYS LINKING SOCIAL STATUS AND HEALTH AMONG OLDER ADULTS

**DOI:** 10.1093/geroni/igz038.3017

**Published:** 2019-11-08

**Authors:** Heather R Farmer, Amy Thierry, Linda A Wray

**Affiliations:** 1 Duke University, Durham, North Carolina, United States; 2 Xavier University of Louisiana, New Orleans, Louisiana, United States; 3 Pennsylvania State University, University Park PA, United States

## Abstract

An abundant literature has documented the social patterning of health, where those with lower social status experience poorer outcomes relative to those with higher status. This symposium examines how social status (e.g., age, race/ethnicity, gender, and SES) impacts various aspects of midlife and older adults’ lives and their psychological and physical health. The research presented in this symposium lend support to utilizing a biopsychosocial framework for understanding mechanisms of health and aging. First, Heather Farmer et al. will explore race and gender differences in elevated C-reactive protein (CRP), a marker of inflammation linked to poor acute and chronic outcomes, using data from the Health and Retirement Study (HRS). Linda Wray and Amy Thierry will use HRS data to test whether race/ethnicity and sex interact to produce unequal outcomes in functional status. Jen Wong et al. will utilize data from the Midlife in the United States (MIDUS) survey to investigate the moderating influences of age, gender, marital status, and social support on caregiving and psychological well-being. Collin Mueller and Heather Farmer will use HRS data to examine how perceptions of unfair treatment are associated with healthcare satisfaction and self-rated health across Black, Latinx, and White subpopulations. Taken together, this work highlights the need for a comprehensive approach to better address physical and mental health disparities over the life course. After attending this session, participants will have a stronger understanding of how social status shapes important outcomes in older adults’ lives and some of the mechanisms responsible for these variations.

